# Entropy of the Land Parcel Mosaic as a Measure of the Degree of Urbanization

**DOI:** 10.3390/e23050543

**Published:** 2021-04-28

**Authors:** Agnieszka Bitner, Marcin Fialkowski

**Affiliations:** 1 Department of Agricultural Land Surveying, Cadaster and Photogrammetry, University of Agriculture in Krakow, Balicka 253A, 30-198 Krakow, Poland; rmbitner@cyf-kr.edu.pl; 2 Institute of Physical Chemistry, Polish Academy of Sciences, Kasprzaka 44/52, 01-224 Warsaw, Poland

**Keywords:** urbanization, land parcel, land division pattern, city growth, spatial analysis, Shannon entropy, Renyi entropy, land fragmentation

## Abstract

Quantifying the urbanization level is an essential yet challenging task in urban studies because of the high complexity of this phenomenon. The urbanization degree has been estimated using a variety of social, economic, and spatial measures. Among the spatial characteristics, the Shannon entropy of the landscape pattern has recently been intensively explored as one of the most effective urbanization indexes. Here, we introduce a new measure of the spatial entropy of land that characterizes its parcel mosaic, the structure resulting from the division of land into cadastral parcels. We calculate the entropies of the parcel areas’ distribution function in different portions of the urban systems. We have established that the Shannon and Renyi entropies $R_{0}$ and $R_{1/2}$ are most effective at differentiating the degree of a spatial organization of the land. Our studies are based on 30 urban systems located in the USA, Australia, and Poland, and three desert areas from Australia. In all the cities, the entropies behave the same as functions of the distance from the center. They attain the lowest values in the city core and reach substantially higher values in suburban areas. Thus, the parcel mosaic entropies provide a spatial characterization of land to measure its urbanization level effectively.

## 1. Introduction

Urban systems have become a subject of extensive studies in recent years. They aim to understand and quantify various spatial, social, economic, and demographic aspects of the urban growth process. In particular, evaluating urban sprawl is an important issue due to its significance in effective urban planning and environmental management. A fair amount of scientific research is focused on the geometric properties of the expanding cities. The main object of interest is the morphology of the landscape pattern obtained by the classification of land concerning the land-use type. Usually, the landscape pattern is determined by the mosaic of built-up and non-built-up pieces of land. Entropy is currently the most widely used method for analyzing the landscape pattern or cartographic maps. It provides an effective tool for quantifying the amount of randomness or information contained in the area under investigation. For this reason, entropy is often employed as a measure of the urban sprawl and urbanization level.

While applying the concept of the thermodynamic entropy to describe urban systems is still problematic [1], spatial entropy [2,3], as a measure of disorder, can be successfully utilized to describe their geometric aspects. Several studies [4,5,6,7,8,9,10,11,12,13] employed entropy as an useful metric to describe the level of organization in various urban spatial structures. Spatial Shannon entropy was used [4] to analyze remote sensing data, and Geographic Information System (GIS) maps to characterize the urban growth patterns. The level of the urban sprawl was also quantified using Renyi entropy [6]. Chen et al. [8] demonstrated that the entropy of the spatial pattern of the land combined with its fractal properties [14,15,16,17,18,19,20] can be successfully applied to characterize the morphology of cities and regions. Spatial conditional entropies were employed [9] as a measure of the urban sprawl values. Huynh et al. utilized [10] the idea of the entropy maximization to characterize the spatial pattern of urban locations. Entropy as a spatial landscape metrics was applied [12] to investigate the nature of the fragmentation of the urban landscape and compactness of the towns.

To measure disorder in the landscape pattern, the concept of the configurational (Boltzmann) entropy was used [11,13]. In the works cited, the Wasserstein metric between the distributions was employed to quantify spatial configurational entropy of the landscape mosaic, and demonstrated that the Wasserstein metric is capable of capturing discrepancies between different spatial configurations. Gudmundsson and Mohajeri [21] have investigated urban street networks of British cities. They introduced entropy measures for quantifying the complexity of street orientations and length variations within planar networks. They found that the entropy and street length increase with distance from the network center. That is, the city center streets are more ordered than those in the outer parts of the city. Interestingly, the second law of thermodynamics was also invoked to explain [22] trends observed in the economy. In the work cited, the author suggested that property follows the universal law of entropy growth, and there is a one-directional bias leading toward increasing its fragmentation.

The cadastral parcel mosaic constitutes the most fundamental land fragmentation structure. It is determined by the boundaries of the lots and covers the whole area of the urban system. The parcel mosaic a geometric attribute of a city that provides useful information about its formation and growth processes. We established [23,24] that the parcel mosaic can form three morphological types that are determined by the shape of the distribution function of the parcel areas, *a*. Three distinct types were found: Highly urbanized core of a city, suburban area, and rural area. In the city core, the probability distribution function possesses a characteristic shape with a single peak located at the parcel size around $10^{3}$ m${}^{2}$, with a tail following the power-law decay with the exponent equal to −2. The core is surrounded by suburban land displaying the log-normal distribution of parcel sizes. The rural area is the outermost part of the urban system and follows the distribution function obeying an inverse power-law with the exponent close to 1. The results [23] suggest that the above classification depends neither on historical conditions nor the land investigated’s geographical location.

Because the cadastral parcel is the elementary building block of the landscape pattern, the parcel mosaic is expected to bear information about the urbanization level. Here, we employ entropy to investigate land parcel mosaic morphology to extract information about its degree of randomness. To achieve this goal, we calculated entropies of the parcel areas’ distribution function in the land under investigation. We established that the Shannon, *S*, and Renyi entropies $R_{0}$ and $R_{1/2}$ are the most effective measures of the amount of randomness of the land. We investigated 30 urban systems located in the USA, Australia, Poland, and low-urbanized areas located in Australia’s desert region. As the main result, we found that the entropies change in the same way with the distance from the city’s origin. They display the lowest values in the city’s central part and significantly higher values in the suburban/rural areas surrounding the city. Our findings prove that the Shannon and Renyi entropies $R_{0}$ and $R_{1/2}$ of the parcel size distribution provide a useful tool for measuring the urbanization level.

## 2. Materials and Methods

### 2.1. Data Sources

Our analysis was based on a collection of Geographical Information System (GIS) cadastral maps in Shapefile format. In the cadastral map, the land parcel (a lot) is the basic spatial unit assigned with a unique parcel number. In the GIS maps the lots are represented by sets of vertexes that determine polygons. The maps contained various types of land parcels, such as built-up and not built-up areas, green areas, public utilities, and industrial areas. Streets and roads were excluded from the analysis. We investigated data covering the whole state of Queensland in Australia (AU) and data for selected counties in the USA: Travis, Tarrant, Williamson, and Harris (TX), Clark (OH), Wake (NC), Marion (IN), and Kern (CA). Additionally, we analyzed one European city (Krakow) located in Poland. The analysis presented in the paper was based on a set of available data. We selected isolated cities for the analysis so that the presence of neighboring towns or villages or other objects like lakes or forests could not distort significantly the parcel mosaic structure. We also investigated data for circular rural areas located in the North-West Region in Queensland (AU). The GIS data for the USA were obtained from publicly accessible sources. The data for Krakow were provided by the City Board of Data Bases in Krakow. The GIS map of Queensland (AU) was obtained courtesy of Professor K. Becek.

### 2.2. Gis Data Processing

#### 2.2.1. Preparation of the Ensembles of the Land Parcels

**Figure 1:**
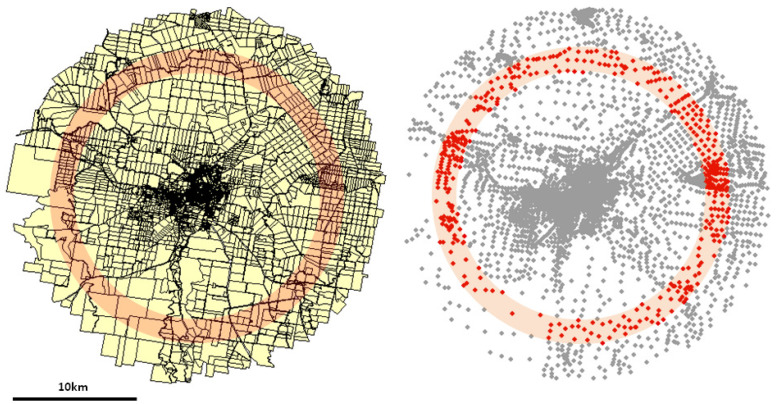
The land parcel mosaic of Warwick (AU) urban system (**left**), and the corresponding arrangement of the centroids of the parcels (**right**). The solid lines represent boundaries of the parcels. The areas of the parcels whose centroids lie within the pinkish ring are collected and used to prepare histogram.

The spatial data concerning the parcels were extracted from the GIS maps and processed using ESRI ArcView software [25]. In the first step, we established the center of the urban system under investigation. In most cases, it was identified with the geometric center of the central business district (CBD). We determined the GIS positions of the parcel (centroid) as its geometric centers of mass. The parcel area was calculated as the area of a polygon determined by the positions of the parcel’s vertexes. Then, the parcels whose centroids were enclosed within a circle of a prescribed radius centered at the city center were exported for further processing. The circle radius was limited by the presence of neighboring towns or villages or other objects like lakes or forests that could distort significantly the parcel mosaic structure. The land was divided into several concentric rings in the next steps. The width of the rings employed for each urban system was 0.5 km. Then the parcels were sorted concerning their distance from the center. The lots whose centroids were included in subsequent rings were collected as an ensemble to prepare the probability distribution (histogram) function. The parcel size distribution functions were calculated for parcels contained in subsequent rings located at various radii from the city center. Importantly, in our approach, we did not take into account the administrative boundaries of the city. All parcels included in the ring, regardless of whether they lie within the city’s administrative boundaries or not, were analyzed. Figure 1 illustrates the data processing procedure described above.

#### 2.2.2. Preparation of the Parcel Area Distribution

The parcel areas in each ensemble analyzed spanned over a wide range of sizes and covered a range of several orders of magnitude. However, bigger areas were sparsely distributed. Thus, to circumvent this problem, to obtain the parcel sizes probability distributions (histograms), the data was split into *N* exponentially spaced bins spanning from the smallest ($a_{min}$) to the greatest ($a_{max}$) value of the parcel area. Such an approach results in binpoints that are equidistant on a logarithmic scale. The set of area values determined the boundaries of the bins $\left\{a_{i}^{b}\right\}$, 0≤i≤N. The positions of the consecutive values of $a_{i}^{b}$ were calculated from the following formula:

(1) $$\begin{array}{ccc} \ln a_{i}^{b} & = & \ln a_{min}+\frac{i}{N}\ln\frac{a_{max}}{a_{min}}, \end{array}$$

The weight, $w_{i}$, of the *i*-th bin (0≤i≤N) was calculated as the difference:

(2) $$\begin{array}{ccc} w_{i} & = & a_{i+1}^{b}-a_{i}^{b}=a_{min}{\left(\frac{a_{max}}{a_{min}}\right)}^{i/N}\left[{\left(\frac{a_{max}}{a_{min}}\right)}^{1/N}-1\right]. \end{array}$$

The weights obey the following relation:

(3) $$\begin{array}{ccc} \sum_{i=0}^{N-1}w_{i} & = & a_{max}-a_{min}. \end{array}$$

The probability associated with the *i*-th bin, $p_{i}$, was obtained as the count in the bin divided by the total number of areas in the ensemble analyzed. To establish the number of bins in the parcel areas’ distribution, we carried out calculations for *N* = 20, 30, 40, and 50. We found that the obtained entropies did not differ much for *N* varying in the range 30–50. For this reason, we chose *N* = 40 as the number of bins to generate the probability distributions.

### 2.3. Calculation of the Entropies

For a given parcel size probability distribution function we calculated the Shannon, *S*, and the Renyi, $R_{q}$, entropies that are defined as

(4) $$\begin{array}{ccc} S & = & -\sum_{i=1}^{n}p_{i}\ln p_{i}, \end{array}$$

and

(5) $$\begin{array}{ccc} R_{q} & = & \frac{1}{1-q}\ln\left(\sum_{i=1}^{n}p_{i}^{q}\right),q\neq 1. \end{array}$$

The parameter *q* is non-extensive parameter. For *q* approaching 1 the Renyi entropy reduces to Shannon entropy. In the case of distributions with non-uniformly spaced bins, *n* is the number of bins, one has to account for different widths of the bins to calculate the entropies [26]. The modified, bin width-weighted formulas for the Shannon and Renyi entropies are the following:

(6) $$\begin{array}{ccc} S & = & -\sum_{i=1}^{n}p_{i}\ln\left(\frac{p_{i}}{w_{i}}\right), \end{array}$$

and

(7) $$\begin{array}{ccc} R_{q} & = & \frac{1}{1-q}\ln\left[\sum_{i=1}^{n}w_{i}{\left(\frac{p_{i}}{w_{i}}\right)}^{q}\right], \end{array}$$

where the weights, $w_{i}$, are given by Equation (2).

The maximum value of the entropy is attained for an uniform distribution of the parcel sizes. Let us denote the maximum value of the distribution of the Shannon and Renyi entropy, respectively, by $S^{sup}$ and $R_{q}^{sup}$. Then, regardless of the binning scheme used, one gets

(8) $$\begin{array}{ccc} S^{sup}=R_{q}^{sup} & = & \ln\left(a_{max}-a_{min}\right). \end{array}$$

We also analyzed a quantity calculated as a difference between the entropy’s maximum value associated with a given distribution and its actual entropy. In the text, this difference is also referred to as the “relative entropy”. The relative Shannon and Renyi entropies are calculated according to the formulas

(9) $$\begin{array}{ccc} S^{sup}-S & = & \ln\sum_{i=1}^{n}p_{i}\ln\left(\frac{p_{i}}{w_{i}^{norm}}\right), \end{array}$$

and

(10) $$\begin{array}{ccc} R_{q}^{sup}-R_{q} & = & -\frac{1}{1-q}\ln\left[\sum_{i=1}^{n}w_{i}^{norm}{\left(\frac{p_{i}}{w_{i}^{norm}}\right)}^{q}\right]. \end{array}$$

Here, the normalized weights, $w_{i}^{norm}$, are defined as

(11) $$\begin{array}{ccc} w_{i}^{norm} & = & \frac{w_{i}}{a_{max}-a_{min}}, \end{array}$$

with the weights $w_{i}$ given by Equation (2). The quantities given by Equations (9) and (10) are always positive, and the lowest possible value is equal to zero. The bigger value of the relative entropy, the more ordered the parcel mosaic is. In the following, for the sake of brevity, we use a common symbol *H* for the Shannon and Renyi entropies.

### 2.4. Analysis of the Dependence of the Entropy on the Distance from the Center of the City

**Figure 2:**
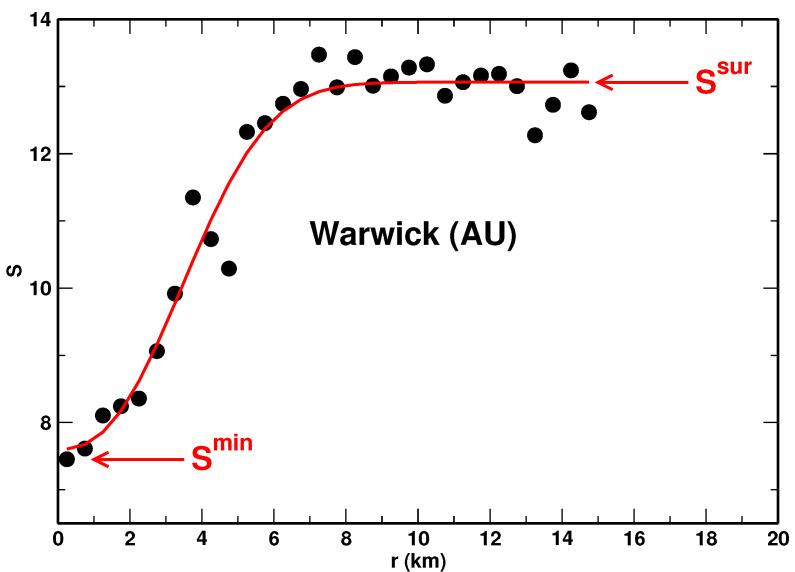
Shannon entropy, *S*, as a function of the distance from the center obtained for the city of Warwick (AU). The solid line represents a fit of Equation (12) to the data. The values of $S^{min}$ and $S^{sur}$ are indicated.

Two quantities were determined based on the obtained dependence of the entropy on the distance from the center of the city. The first one was the entropy’s value, $H^{sur}$, observed in the area surrounding the city. $H^{sur}$ was calculated only for the urban systems for which the entropy as a function of *r* flatted out to reach an apparent plateau. In our approach, $H^{sur}$ was obtained as a fitting parameter. We employed the least-squares fit of the following stretched-exponential function to the data:

(12) $$\begin{array}{ccc} H\left(r\right) & = & H^{sur}-\Delta_{H}\exp\left[-{\left(\frac{r}{\rho }\right)}^{\alpha }\right], \end{array}$$

with $H^{sur}$, $\Delta_{H}$, ρ, and α being fitting parameters. The fitting procedure allowed us to avoid arbitrariness in selecting the data points for calculating the plateau level. Moreover, the fitting provided the statistical uncertainty of $H^{sur}$. The second quantity was the minimum entropy, $H^{min}$, observed within the city, typically in its central portion (core). It was determined as the lowest value in the set $H\left(r_{i}\right)$. The meaning of the quantities $H^{min}$ and $H^{sur}$ is explained in Figure 2, where the dependence of the Shannon entropy on the distance *r* is plotted for the city of Warwick (AU).

## 3. Results and Discussion

We investigated 9 urban systems located in the USA, 20 in Australia, and one in Poland. For each city, we calculated parcel size distribution functions for parcels contained in concentring circular rings located at various radii from the city center. We investigated how different entropies of the distribution function change with the distance *r* from the center. Our purpose was to find a new measure of urbanization level. We have chosen two representative entropies: Shannon (applicable to systems with extensive property) and Renyi entropy (for non-extensive systems). Surprisingly both can be used as a measure of the level of land urbanization. We analyzed the Renyi entropies, $R_{q}$, for *q* = −2, −1/2, 0, 1/2, and 2, and the Shannon entropy, *S*. To calculate $R_{q}$ and *S*, the bin width-weighted formulas given by Equations (6) and (7), respectively, were employed.

**Figure 3:**
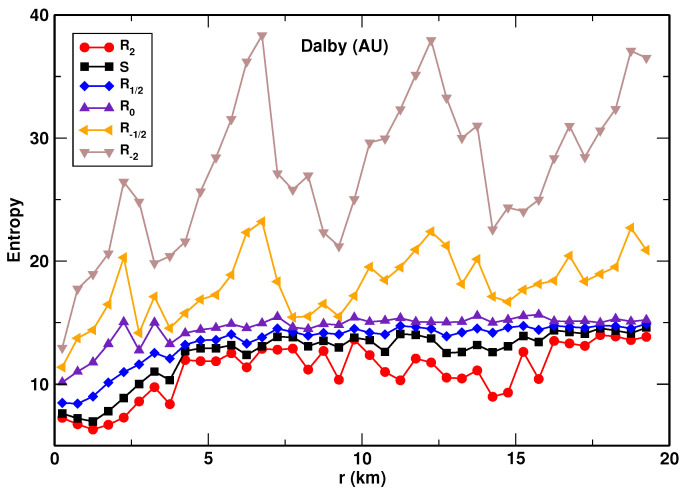
The entropies $R_{-2}$, $R_{-1/2}$, $R_{0}$, $R_{1/2}$, *S*, and $R_{2}$ plotted as functions of the distance from the center of a city of Dalby (AU).

**Figure 4:**
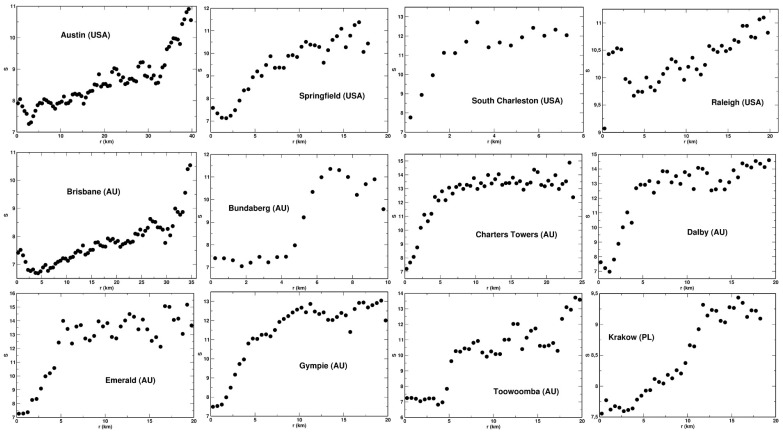
Shannon entropy plotted as a function of *r* for selected urban systems.

**Figure 5:**
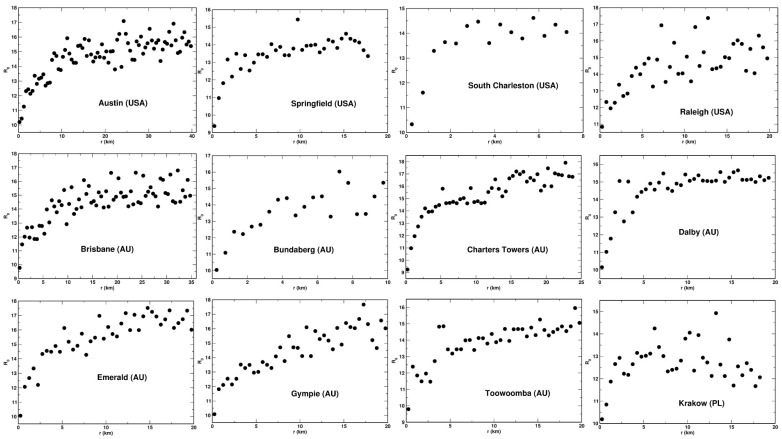
Renyi entropy, $R_{0}$, plotted as a function of *r* for selected urban systems.

**Figure 6:**
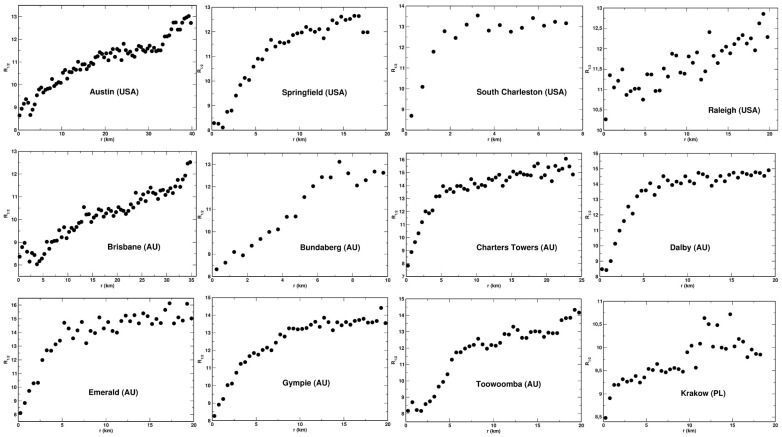
Renyi entropy, $R_{1/2}$, plotted as a function of *r* for selected urban systems.

We established that for each system analyzed, the values of the entropies obey the following relationship: $R_{-2}>R_{-1/2}>R_{0}>R_{1/2}>S>R_{2}$. This is shown for the example of the city of Dlaby (AU) in Figure 3, where the five entropies are plotted a function of the distance, *r*, from the center. We also found that, among the entropies analyzed, the Renyi entropies with the indexes *q* = 0 and 1/2, and the Shannon entropy display the most regular behavior as the functions of *r*. That is, they are the least scattered and exhibit best to determine variability with *r*. This feature of the entropies is also demonstrated in Figure 3. For this reason, we selected the three entropies for detailed analysis: *S*, $R_{0}$, and $R_{1/2}$. These entropies as the function of the distance, *r*, are presented for 12 selected urban system in Figure 4, Figure 5 and Figure 6, respectively. We found that the Shannon entropy changes with *r* similarly for all the cities analyzed. Generally, it increases with the distance from the center to reach a plateau. In some cases (e.g., Brisbane or Austin), it has a minimum in the center’s vicinity. Moreover, for some cities, the entropy grew with *r* without reaching a plateau. Qualitatively, the same dependence on *r* exhibits the Renyi entropy $R_{1/2}$, as shown in Figure 6. As to the Renyi entropy $R_{0}$, it behaves in quite a similar way to *S* and $R_{1/2}$ as a function of *r*. It is, however, generally more scattered. What also makes $R_{0}$ different from the other two is the lack of the minimum. That is, $R_{0}$ exhibits a monotonic growth with the distance from the center.

**Figure 7:**
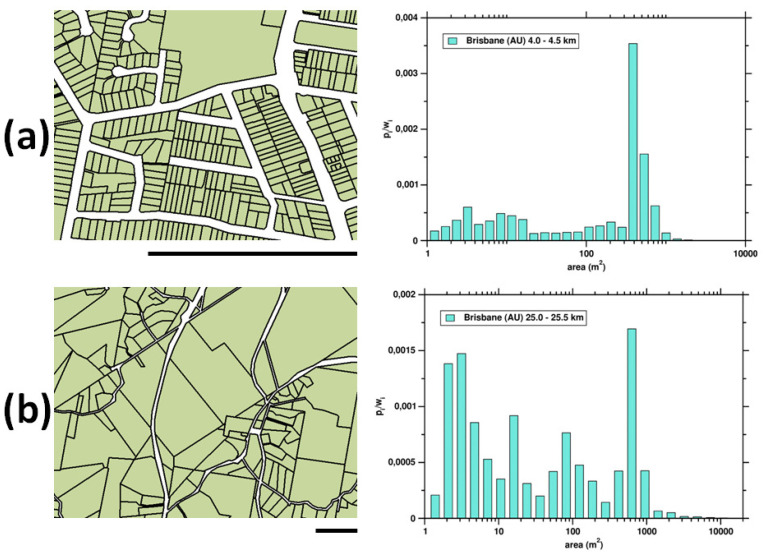
Two samples of the land parcel mosaic observed in (**a**) 4 km from the center of Brisbane, and (**b**) in the suburban region of Brisbane at the distance of 25 km from the center. In both cases the scale bar is 500 m. The white color in the maps represents streets that are excluded from the analysis. The corresponding parcel area probability distribution functions are shown on the (**right**).

All the entropies analyzed displayed the lowest values in cities’ centers and grow with the distance from the center to reach maximum value in the suburban or rural areas surrounding the city. The city cores represent the highest urbanization level that is also encoded in their spatial structure. In particular, the spatial organization is reflected in the morphology of the land parcel mosaic. A regular street grid organizes the city center’s space, and land parcels of some characteristic size prevail. In the suburban and rural areas, the land is much less organized. In terms of the land fragmentation pattern, the parcels areas’ distribution is more spread out compared to that observed in the center. This difference is illustrated in Figure 7, where the land parcel size distributions observed in the center of Brisbane and in its distant suburbian areas are presented along with the corresponding parcel mosaic samples.

As mentioned, in some cases, the entropies *S* and $R_{1/2}$ exhibit a shallow minimum in some distance from the city center. This can be because of the choice of the specific location of the origin. However, the maps of the city cores suggest other, more plausible explanation of this fact. Namely, it can be attributed to the presence of parks, large squares, and public facilities. They introduce some disorder into the spatial structure and gives rise to the increase of the entropies. A more regular form of the land fragmentation pattern is observed at a certain distance from the city center.

**Figure 8:**
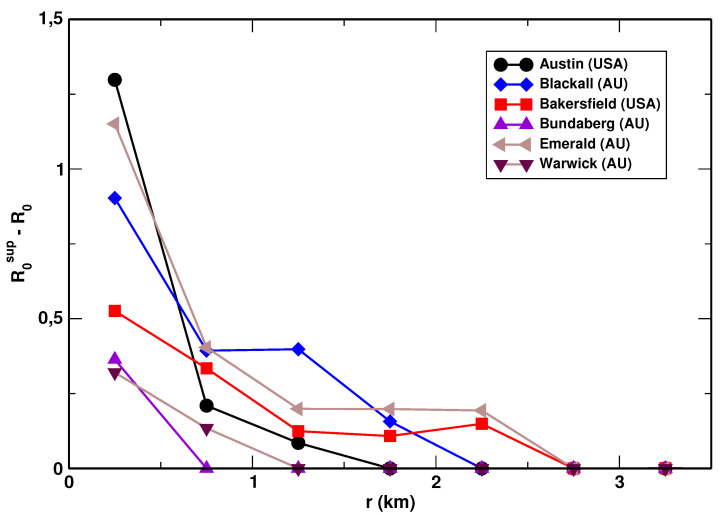
The dependence of the relative entropy $R_{0}^{sup}-R_{0}$ as a function of the distance from the center plotted for selected cities.

Besides the Shannon and Renyi $R_{0}$ and $R_{1/2}$ entropies, we investigated the relative entropies defined by defined by Equations (9) and (10). We found that $R_{0}^{sup}-R_{0}$ possesses properties that are best suitable for the determination of the urbanization level. We found that the relative entropy $R_{0}$ exhibited the same behavior for most of the urban systems analyzed. The dependence of the relative $R_{0}$ entropy for selected cities is shown in Figure 8. It attains some non-zero value at the origin and rapidly drops to zero. We did not observe values of $R_{0}^{sup}-R_{0}$ bigger than 10${}^{-3}$ for *r* > 3.0 km. There were three urban systems for which the relative $R_{0}$ entropy was equal to zero for all distances from the center: Maryborough (AU), Toowoomba (AU), and Krakow (PL). Very small values were observed also for Houston (USA). The observed properties of $R_{0}^{sup}-R_{0}$ can be explained in view of Equation (10). Namely, for q=0 the relative Renyi entropy becomes a function of the fraction ϕ of the distribution domain for which $p_{i}>0$, viz. $R_{0}^{sup}-R_{0}=-\ln\phi$. In the highly organized city cores the parcel area distribution generally displays some gaps (empty bins) in its domain, which yields ϕ<1. When the distance from the core increases, the parcel size distribution becomes more uniform, the gaps are filled, and the relative entropy rapidly reaches the value of zero. Thus, the positive value of $R_{0}^{sup}-R_{0}$ is characteristic of spatially well-organized city centers.

For each system, we determined the minimum entropies $H^{min}$ along with the corresponding values of $H^{sur}$. The results of the analysis are summarized in Table 1. In some cases, the values of $H^{sur}$ were impossible to determine because the dependence of $H\left(r\right)$ did not exhibit a clear plateau. In the last column, the maximal values of the relative entropies, calculated as $R_{0}^{sur}-R_{0}^{min}$, are listed.

It follows from Table 1 that the minimal Shannon entropy attains quite similar values for all cities. The same is true for the Renyi entropies $R_{0}^{min}$ and $R_{1/2}^{min}$. Moreover, for each country, there is a significant difference between the values of $H^{min}$ and the values $H^{sur}$ observed in the surrounding suburban areas. Table 2 shows average values of the entropies analyzed calculated separately for the USA and Australia (data for Krakow (PL) are also presented), and the minimum entropies averaged for the whole set of cities studied. Note that there are some differences between the average values, $\langle H^{min}\rangle$, obtained for each country. However, the data presented in Table 2 indicate that the value of 10.0 is—within statistical uncertainty—an constant value for the average $R_{0}^{min}$ in all areas studied.

**Figure 9:**
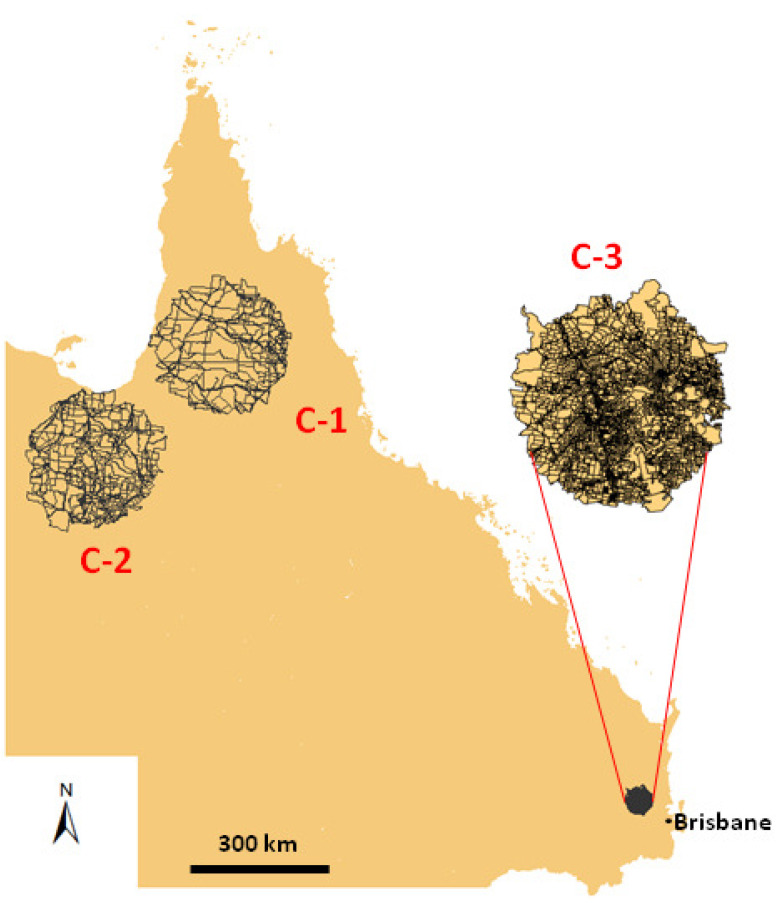
Location of the areas C-1, C-2, and C-3 on the map of Queensland in Australia. The solid lines represent boundaries of the land parcels.

To further explore the land parcel mosaic properties, we also investigated three non-urbanized areas located in the desert regions in Queensland, Australia. They are denoted as C-1, C-2, and C-3, have forms of circles. They are remote from urban systems and do not contain any cities or bigger settlements. Locations of these areas on the map of Queensland are visualized in Figure 9. The dots indicate the centroids of the land parcels. For each area we determined Shannon and Renyi entropies $R_{0}$ and $R_{1/2}$. Moreover, the value of the relative Renyi entropy, $R_{0}^{sup}-R_{0}$ was calculated for each land. The results are summarized in Table 3. As seen, all the areas display relatively high values of the entropies compared to the values of $H^{min}$ observed in the urban systems that are listed in Table 1. However, they overlap with the corresponding values of the $H^{sur}$ entropies. The results show that, although all three areas represent similar low-populated low-developed rural land, they exhibit different urbanization (organization) levels. Based on the Shannon and Renyi entropies’ values, one can conclude that the region C-1 is less developed than C-2, and the region C-2 than C-3.

**Figure 10:**
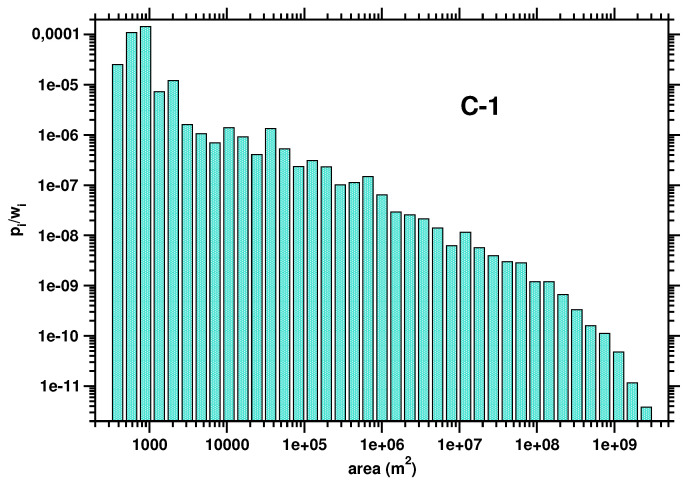
Parcel area probability distribution function calculated for the area C-1.

We found that the relative Renyi entropy, $R_{0}^{sup}-R_{0}$, is equal to zero for all the regions under investigation. This result is not surprising as the areas C-1, C-2, and C-3 represent an early stage of land development. The parcel mosaic in such areas results from a very initial cadastral division that can be modeled by the process of random partitioning of a plane [23,27,28]. In this process, which is also referred to as the fenced-off process, the plane is subsequently subdivided by straight lines randomly oriented and positioned. Each line divides the area into two parts. The smaller part is selected as a parcel, while the larger one undergoes further fragmentation. This land division process leads to an inverse power law distribution of the parcel areas, $f\left(a\right)∼a^{-\beta }$, with the exponent β close to 1. The parcel area distribution for the region C-1 is presented in the double logarithmic scale in Figure 10. As seen, the distribution follows with a good approximation of the inverse power law function. The exponent was found to be β=0.88±0.03. The area distributions for the regions C-2 and C-3 have quite similar shapes.

**Figure 11:**
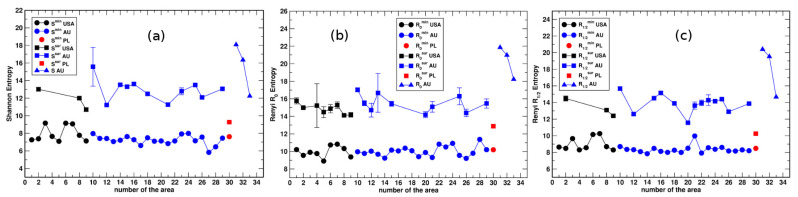
Summary of results for the urban systems and three desert areas for (**a**) *S*, (**b**) $R_{0}$, and (**c**) $R_{1/2}$. The numbers on the abscissa correspond to the numbers of the urban systems and the desert areas that are given in Table 1 and Table 3.

Figure 11 summarizes results for all the urban systems and the non-urbanized areas investigated. The results presented demonstrate that both the Shannon and Renyi entropies of the parcel area distribution provide a spatial characterization of land that can be used to quantify the urbanization level. First, the data show that the value of the minimal Shannon entropy attained within the urban system (Figure 11a) is quite similar for all the cities studied. Significantly, they are clearly separated from the values of $H^{min}$. The same is also true for the minimal Renyi entropies $R_{0}^{min}$ (Figure 11b) and $R_{1/2}^{min}$ (Figure 11c). Second, the values of $S^{sur}$ observed in the suburban or rural areas surrounding the cities vary in some range, reflecting differences in the urbanization degrees. The $H^{sub}$ entropies are calculated for an ensemble of parcel areas collected in a piece of land that is uniform with respect to the urbanization level. Thus, they can be compared to the corresponding values of the entropies obtained for the circles C-1, C-2, and C-3. The highest values of the Shannon entropy were observed in the non-developed areas located in the desert. In contrast, the smallest ones were found in the suburban land surrounding well-developed urban systems. As demonstrated in Figure 11b,c, the same behavior exhibit also the Renyi entropies $R_{0}$ and $R_{1/2}$.

## 4. Conclusions and Discussion

Urbanization is a process by which a given area and its inhabitants acquire urban characteristics. The process transforms the environment and leaves behind a characteristic spatial structure as a kind of its morphological fingerprint. Here, a morphological fingerprint of the urbanization process is a mosaic formed by land parcels. Parcel boundaries are the most solid form of human settlements. We believe that changes of the morphological structure of land division are the first sign of the process of spatial urbanization. This is because land division starts further transformations, functional changes and land settlement. The paper “Universal rules for fragmentation of land by humans” [23] was the first to point out that the structure of cadastral parcels might be an indicator of the level of land urbanization.

In this paper a new measure of urbanization level, the spatial entropy of parcel mosaic, was introduced. We have applied Shannon and Renyi entropies to investigate the land parcel mosaic morphology. In our approach, we have calculated entropies of the distribution function of the parcel areas. We have found that the Shannon, *S*, and Renyi entropies $R_{0}$ and $R_{1/2}$ are the best at differentiating the level of the spatial organization of the land. We have studied 30 urban systems located in the USA, Australia, Poland, and three low-urbanized areas situated in Australia’s desert. For all the cities analyzed, it has been found that the entropies display the same behavior as functions of the distance, *r*, from the city center. They attain the lowest values in the central oldest part of the city and grow with *r* to reach substantially higher values in the suburban or rural areas surrounding the city. The highest values of the entropies have been observed in the desert-like regions. Remarkably, the minimum value of a given entropy observed in the city core takes similar values in all the urban systems analyzed. Thus, we conclude that the Shannon and Renyi entropies $R_{0}$ and $R_{1/2}$ of the parcel size distribution provide a robust spatial characterization of land that can be used as a measure of its urbanization level.

Our studies demonstrate that cadastral maps are a reliable source of information about large-scale spatial phenomena. Information about the degree of urbanization enables a more optimal use of the area under study. It facilitates the creation of strategies for sustainable development of the area and using it for industrial or commercial investments, for investing in hotels or housing. Determination of parcel mosaic entropies enables better land management.

## Figures and Tables

**Table 1:** Results of the analysis performed for 30 urban systems located in the USA, Australia (AU), and Poland (PL). The dash represents that the value of a given quantity was impossible to establish.

No.	City	Country	Population	$S^{\min}$	$S^{\mathrm{sur}}$	$R_{0}^{\min}$	$R_{0}^{\mathrm{sur}}$	$R_{1/2}^{\min}$	$R_{1/2}^{\mathrm{sur}}$	$R_{0}^{\sup}-R_{0}^{\min}$
1	Austin, TX		656,562	7.26	-	10.21	15.77 ± 0.34	8.64	-	1.30
2	Bakersfield, CA		247,057	7.38	13.01 ± 0.23	9.55	14.99 ± 0.06	8.48	14.48 ± 0.31	0.90
3	Fort Worth, TX		534,694	9.16	-	9.90	-	9.66	-	0.77
4	Georgetown, TX		28,339	7.65	-	9.76	15.23 ± 2.5	8.30	-	0.73
5	Houston, TX	USA	1,953,631	7.09	-	8.90	14.48 ± 0.62	8.56	-	0.03
6	Indianapolis, IN		807,459	9.15	-	10.74	14.88 ± 0.47	10.15	-	0.63
7	Raleigh, NC		276,093	9.07	-	10.83	15.28 ± 0.37	10.26	-	1.34
8	S. Charleston, OH		1850	7.77	12.00 ± 0.15	10.33	14.12 ± 0.11	8.69	13.08 ± 0.09	0.14
9	Springfield, OH		8990	7.13	10.70 ± 0.20	9.37	14.18 ± 0.26	8.29	12.40 ± 0.10	0.16
10	Blackall		1662	7.97	15.57 ± 2.20	9.96	17.03 ± 0.24	8.70	15.68 ± 0.24	0.53
11	Brisbane		958,504	7.42	-	9.77	15.50 ± 0.28	8.36	-	0.00
12	Bundaberg		45,873	7.41	11.22 ± 0.09	10.03	14.71 ± 0.78	8.32	12.61 ± 0.15	0.36
13	Cairns		125,327	7.06	-	9.68	16.67 ± 2.2	8.09	-	1.36
14	Charters Towers		8846	7.21	13.52 ± 0.09	9.24	-	7.84	-	0.97
15	Dalby		10,215	7.63	13.30 ± 0.12	10.15	15.44 ± 0.29	8.48	14.52 ± 0.08	0.11
16	Emerald		13,523	7.27	13.61 ± 0.15	10.06	-	8.12	15.15 ± 0.20	1.15
17	Gladstone		28,548	6.61	-	10.37	-	8.00	-	0.90
18	Gympie		16,000	7.49	12.49 ± 0.09	10.08	-	8.26	13.89 ± 0.13	0.35
19	Innisfail	AU	8394	7.10	-	9.40	-	7.99	-	0.32
20	Ipswich		135,791	7.13	-	9.88	14.19 ± 0.34	8.50	11.57 ± 0.07	0.48
21	Mackay		79,949	6.83	11.26 ± 0.18	9.31	15.09 ± 0.61	9.97	13.63 ± 0.43	0.55
22	Maryborough		25,635	7.13	-	10.83	-	7.92	13.95 ± 0.34	0.00
23	Nanango		4500	7.93	12.78 ± 0.39	10.51	-	8.56	14.28 ± 0.64	0.29
24	Rockhampton		59,755	7.99	-	10.92	-	8.38	14.15 ± 0.15	0.63
25	Roma		6736	7.15	13.50 ± 0.12	9.55	16.29 ± 0.97	8.60	14.40 ± 0.16	0.70
26	Stanthorpe		10,592	7.57	12.09 ± 0.15	9.21	14.39 ± 0.42	8.17	12.89 ± 0.13	0.59
27	Toowoomba		94,189	5.83	-	9.79	-	8.16	-	0.00
28	Townsville		98,075	6.46	-	11.37	-	8.30	-	0.22
29	Warwick		21,564	7.45	13.06 ± 0.11	10.20	15.47 ± 0.52	8.20	13.86 ± 0.08	0.32
30	Krakow	PL	734,400	7.62	9.27 ± 0.05	10.19	12.88 ± 0.14	8.48	10.26 ± 0.08	0.00

**Table 2:** Average values of *S*, $R_{0}^{min}$, and $R_{1/2}^{min}$.

Country	$\langle S^{\min}\rangle$	$\langle R_{0}^{\min}\rangle$	$\langle R_{1/2}^{\min}\rangle$
USA	7.96 ± 0.30	9.95 ± 0.21	9.00 ± 0.26
AU	7.23 ± 0.12	10.02 ± 0.13	8.35 ± 0.10
PL	7.62	10.19	8.48
USA+AU+PL	7.46 ± 0.13	10.00 ± 0.11	8.55 ± 0.12

**Table 3:** The results for three circular regions located in the desert areas in Queensland, Australia. The positions of the centers are given in the Geographic Coordinate System GCS_GDA_1994.

No.	Region	Coordinates of the Center (X, Y)	Radius (km)	*S*	$R_{0}$	$R_{1/2}$
31	C-1	142.918516, −16.651888	150	18.08	21.86	20.40
32	C-2	139.963044, −19.201577	150	16.35	20.98	19.53
33	C-3	152.439683, −27.021106	30	12.24	18.23	14.66

## Data Availability

Not applicable.
